# Disruption of G-Protein γ_5_ Subtype Causes Embryonic Lethality in Mice

**DOI:** 10.1371/journal.pone.0090970

**Published:** 2014-03-05

**Authors:** Anne M. Moon, Anna M. Stauffer, William F. Schwindinger, Kathy Sheridan, Ashley Firment, Janet D. Robishaw

**Affiliations:** The Weis Center for Research, Geisinger Clinic, Danville, Pennsylvania, United States of America; Cincinnati Children’s Hospital Medical Center, United States of America

## Abstract

Heterotrimeric G-proteins modulate many processes essential for embryonic development including cellular proliferation, migration, differentiation, and survival. Although most research has focused on identifying the roles of the various αsubtypes, there is growing recognition that similarly divergent βγ dimers also regulate these processes. In this paper, we show that targeted disruption of the mouse *Gng5* gene encoding the γ_5_ subtype produces embryonic lethality associated with severe head and heart defects. Collectively, these results add to a growing body of data that identify critical roles for the γ subunits in directing the assembly of functionally distinct G-αβγ trimers that are responsible for regulating diverse biological processes. Specifically, the finding that loss of the G-γ_5_ subtype is associated with a reduced number of cardiac precursor cells not only provides a causal basis for the mouse phenotype but also raises the possibility that G-βγ_5_ dependent signaling contributes to the pathogenesis of human congenital heart problems.

## Introduction

Diverse types of receptors (*ie*, G-protein-coupled [1]–[3], frizzled [4]–[5], smoothened [6]–[8], integrin [9]–[10], and growth factor [11]–[12] receptors) converge on heterotrimeric G-proteins to coordinate embryonic development. Following activation of the upstream receptor, the G-protein undergoes conformational rearrangements to produce two signaling moieties – a GTP-bound α subunit and a functional βγ dimer – that initiate bifurcating signaling cascades to yield the appropriate cellular response(s) [13]–[15]. Based on the known number of G-subunit genes [16], there is the potential to generate hundreds of distinct G-αβγ combinations that could operate in the context of embryonic development. However, identifying which particular G-αβγ heterotrimers actually exist *in vivo* and how they function in various developmental processes has been challenging.

Gene targeted disruption offers a powerful approach to answer these questions. Because the functions of G-αβγ heterotrimers are traditionally ascribed to the α subtypes, targeted disruption of all 16 *Gna* genes has been performed in mice [17]. Loss of the *Gnas* gene produces gastrulation defects [18], while ablation of the *Gna13* gene produces embryonic lethality associated with vascular problems [19]. Likewise, combinatorial disruption of the related *Gna11* and *Gnaq* genes causes cardiac hypoplasia and perinatal lethality [20], while coincident loss of all three *Gnai* genes produces pups with skeletal defects [21]. In contrast, much less is known regarding the functions of the individual G-β and γ subtypes. Targeted disruption of two of the five *Gnb* genes has been carried out in mice [22]–[23], with loss of the *Gnb1* gene producing partial embryonic lethality associated with incomplete closure of the neural tube [22]. More recently, genetic inactivation of four of the twelve *Gng* genes has been performed [24]–[29]. Although no developmental defects are reported, individual disruption of the *Gngt1*, *Gng3*, *Gng7*, and *Gng13* genes produce distinct phenotypes indicating their requisite roles in specific physiological processes that cannot be substituted by other family members [25]–[29]. This supports the notion that functional specificity of G-βγ dimers not only exists but further suggests that such specificity is attributable to the numerous and structurally diverse γ component [24], [30], [31].

The *Gng5* gene encoding the G-γ_5_ subtype shows many interesting features suggesting an important role in embryonic development. The *Gng5* transcript is highly expressed in the anterior portion of the embryo giving rise to brain and heart structures (www.genat.org). Moreover, the *Gng5* transcript is enriched in neural progenitor cells in both embryonic and adult brain [32]–[33]. Finally, the G-γ_5_ protein is present in focal adhesions important for regulating cellular adhesion, proliferation, and migration [34]. This paper shows for the first time that targeted disruption of the *Gng5* gene causes complete embryonic lethality. Mutant embryos are readily identifiable by their abnormal headfolds, hypoplastic pharyngeal arches, and severe cardiac defects. These findings are novel in several respects. First, they add to a growing body of evidence that the G-γ subtypes are not functionally interchangeable in the context of the animal. Second, they reveal a critical requirement for the G-γ_5_ subtype in the second wave of cardiac development contributing to the formation of the right ventricle and outflow tract. Since the cardiac defects resulting from loss of the G-γ_5_ subtype are much more severe than individual or combinatorial disruption of any of the G-α subtypes [17]–[21], these results suggest a separate requirement for G-βγ_5_ signaling above and beyond that of any G-α pathway in this process. Although the mechanism is still being investigated, we hypothesize that G-βγ_5_ signaling may represent a point of convergence for G-protein-coupled, integrin, and fibroblast growth factor receptor signaling pathways that are critical for the expansion or survival of cardiac progenitor cells within the second heart field. This knowledge could contribute to a better understanding of human congenital heart defects arising from abnormalities within this region.

## Materials and Methods

### Production of Gng5 Mutant Mice

**Figure 1:**
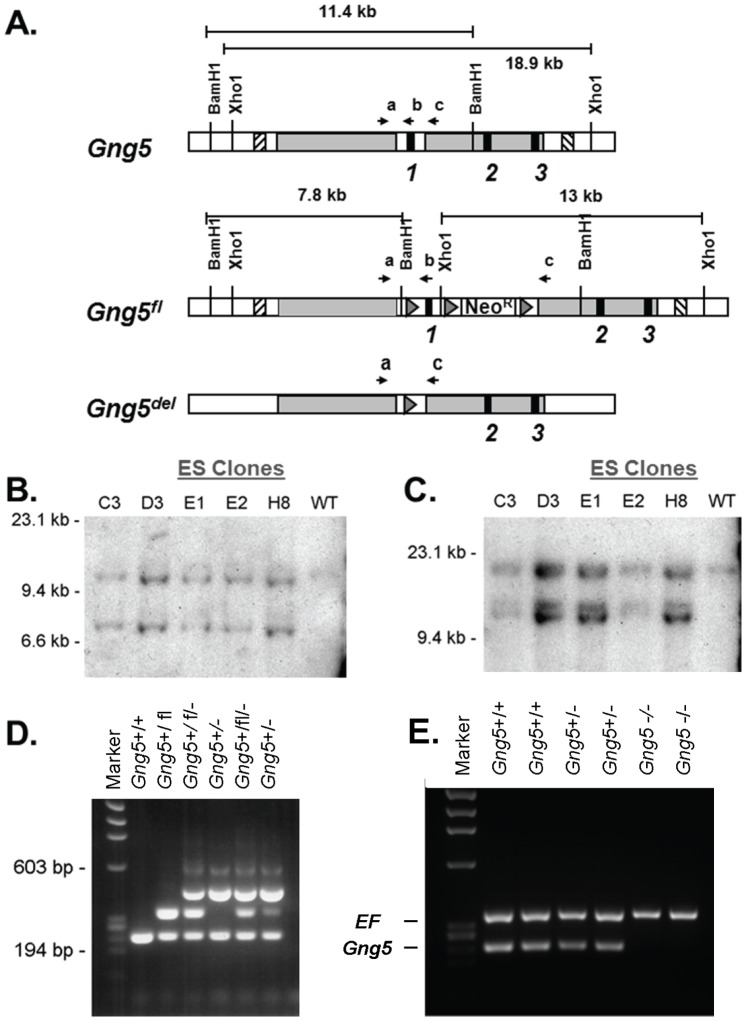
Production of *Gng5* mutant mice. **A.** Wild type *Gng5* allele (top bar) illustrating the three *Gng5* exons (black boxes), homology arms of the targeting vector (grey boxes) and 5′ and 3′ probes for Southern blotting (striped boxes). Floxed *Gng5* allele (middle bar) illustrating insertion of loxP sites (triangles) and neomycin resistance cassette (Neo^R^). Deleted *Gng5* allele (bottom bar) illustrating deletion of sequence between 1^st^ and 3^rd^ loxP site including the first exon of *Gng5* and *Neo^R^* cassette. Also illustrated are expected sizes of fragments following digestion with BamHI (B) or XhoI (X), along with the primers used for PCR (**a,b,c**). Primer sequences can be found in Table S1. **B.** Southern blot of DNA prepared from five properly-targeted ES cell clones (C3-H8) and one wild type clone (WT). DNA was digested with BamHI and probed with the 5′-probes. Properly targeted clones displayed both the 7.8 kb floxed allele and the 11.4 kb wild type allele. **C.** DNA from these same clones was digested with XhoI and probed with the 3′-probe. Properly targeted clones displayed both the 13 kb floxed allele and the 18.9 kb wild type allele. **D.** PCR analysis of DNA from tail biopsy of a wild mouse *Gng5* ^+/+^, a floxed mouse *Gng5* ^+/fl^, and offspring of a cross between a floxed mouse and an Tg(*EIIa-cre*) mouse showing varying degrees of recombination *Gng5* ^+/fl:del^ or *Gng5^+/del^*. The first lane is the φX HaeIII molecular weight marker. Primers **a** and **b** produce a 241-bp band from the wild type (+) allele or a 333-bp band from the floxed (fl) allele. Primers **a** and **c** produce a 454-bp band from the deleted (−) allele resulting from recombination between the 1^st^ and 3^rd^ loxP site. **E.** RT-PCR of *Gng5* (primers **d** and **e**) and eukaryotic elongation factor (EF) from two wild type (*Gng5* ^+/+^), two heterozygous (*Gng5* ^+/−^), and two homozygous knockout (*Gng5* ^−/−^) embryos, confirming the absence of *Gng5* mRNA transcript in knockout embryos.

To provide the potential for conditional inactivation, the targeting vector was designed to add a *loxP* site upstream of the first exon and to introduce a *neo^r^* selectable marker flanked by *loxP* sites in the first intron of the mouse *Gng5* gene (Fig. 1A). After electroporation, embryonic stem cells containing the floxed allele were injected into blastocysts to create chimeric mice (Fig. 1B,C). Following germline transmission, the mice carrying the floxed allele (*Gng5* ^+/fl^) were obtained on a contractual basis from Caliper Life Sciences, Cranbury, NJ (Fig. 1D). Finally, after breeding to Tg(*EIIa-Cre)* mice, the mice containing the globally disrupted allele (*Gng5* ^+/del^) were produced (Fig. 1D) and loss of *Gng5* expression was confirmed (Fig. 1E). Prior to characterization, the *Gng5* ^+/del^ mice were backcrossed to C57Bl6J mice for >10 generations to minimize genetic variability. Both Tg(*EIIa-Cre)* and C57/Bl6J mice were obtained from Jackson Laboratories (Bar Harbor, ME).

### Ethics Statement

Animal importation and usage was approved by the Geisinger Institutional Animal Care and Use Committee (protocol number: 109-11; approval date: 9/25/13), and was performed in strict accordance with NIH recommendations published in the Guide for the Care and Use of Laboratory Animals.

### Genotyping

*Gng5* ^+/−^ mice were intercrossed to produce the three experimental groups (*Gng5^+/+^*, *Gng5* ^+/−^, *Gng5* ^−/−^). For collection of embryos at specific stages, timed matings were performed, with the appearance of a vaginal plug marking embryonic day, e0.5. For genotyping of animals, genomic DNA was prepared from the yolk sac of embryos, or tails of pups. Subsequently, PCR amplification of the wild type or deleted fragment was performed with two primers that flanked the *loxP* site upstream of *Gng5*, and a third primer lying just downstream of the *neo^R^* cassette integration site (Table S1), using a PTC-100 programmable thermal cycler (MJ Research, St. Bruno, Canada).

### Quantitative (q)PCR Analysis

RNA was prepared from yolk sacs, embryos, or micro-dissected, pharyngeal tissues using a Trizol-based procedure (Invitrogen, Carlsbad, CA). First-strand cDNA was prepared from 1 µg of total RNA primed with random hexamers in a reaction catalyzed by MMLV reverse transcriptase (Promega, Madison, WI). For qPCR analysis, either embryonic cDNA, or a developmental cDNA Panel (Clontech, Palo Alto, CA) was used as template to amplify G-γ transcripts and other genes of interest. For this purpose, primers were designed to span intron junctions and their sequences can be found in Table S1. All reactions were performed with iQ Sybr Green Supermix and run on the iCycler device (BioRad, Hercules, CA). Relative gene expression was calculated using the 2(−DeltaDeltaC(T)) method [35]. All expression analyses were performed in triplicate and significant differences identified by Student t-test.

### RNA in situ Hybridization and RNAscope Analyses

Embryos were fixed in 4% paraformaldehyde, dehydrated with ethanol, and paraffin-embedded. Subsequently, embryos were sectioned (6- µm), processed for RNA *in situ* hybridization, or stained with Hematoxylin and Eosin. To detect expression of the *Gng5* and fibroblast growth factor 8 (*Fgf8)* genes, RNA *in situ* hybridization was performed on sectioned embryos using the RNAscope Brown 2.0, *In Situ* hybridization kit (Advanced Cell Diagnostics, Hayward, CA). For this purpose, the *Gng5* probe was designed to transcript NM_010318.2 (nt 2–474), while the *Fgf8* probe was designed to transcript NM_010205 (nt 317–1008). Prior to *in situ* hybridization, the slides were baked at 58°C (1-hour) to soften the paraffin; cleared in xylene (2×5-minutes), rinsed in 100% ethanol (2×3-minutes), and then air dried. After circling tissues with a hydrophobic barrier pen, the RNAscope protocol was performed according to manufacturer’s recommendation (Advanced Cell Diagnostics), with the following exceptions: Pretreatment 2 was performed at 95°C for 10-minutes; Pretreatment 3 was diluted 1∶5 with 1X phosphate-buffered saline and carried out at 40°C for 30-minutes; and Ammonia wash was extended for increased contrast between DAB staining and tissue. Subsequently, the slides were hybridized with test probes (*Fgf8* or *Gng5*) at 40°C for 2-hours, using the following incubation conditions: Amp 1 at 40°C for 30-min; Amp 2 at 40°C for 15-min; Amp 3 at 40°C for 30-min; Amp 4 at 40°C for 15-min; Amp 5 at room temperature for 30-min; and Amp 6 at room temperature for 15-min. All incubations were carried out using the HybEZ hybridization oven with humidifying chamber (Advanced Cell Diagnostics). Between each step, the slides were washed in the provided wash buffer (2×2-minutes). Finally, the colorimetric reaction was performed with the 1∶1 DAB solution (equal volumes of DAB-A and DAB-B were mixed directly before addition to the tissue) at room temperature for 10-minutes. The slides were rinsed with water, stained with 50∶50 Myers Hematoxylin/H_2_O, rinsed with water, and then rinsed again with 0.01% ammonia water. After drying tissue by dehydration with 70% ethanol (2-minutes), rinsing with 100% ethanol (2×2-minutes), and clearing with xylene (5-minutes), the coverslips were mounted with cytoseal for microscopic assessments.

### Proliferation and TUNEL Analyses

Somite/stage-matched embryos were processed as described previously [36]. To prepare cryosections, embryos were fixed in 4% paraformaldehyde and then protected in a sequential series of 10, 20 and 30% sucrose/PBS solutions, oriented in OCT (Tissue Tek) filled molds, frozen, and then cut into 10-µm sections. Subsequently, sections were washed with PBS, blocked in 2% bovine serum albumin with 0.5% Triton-X100 and incubated overnight with anti-phosphohistone antibody (anti-HH3; 1∶500; Millipore #06-570). After washing, sections were washed, blocked, and incubated with AlexaFluor 488 conjugated secondary antibody (1∶500; Jackson ImmunoResearch #711-545-152). Simultaneous TUNEL was performed by adding TMR Red *in situ* cell death detection reagents (Roche) to the secondary antibody incubation. Sections were preserved in Vectashield anti-fading reagent (Vector Laboratories) and captured by confocal microscopic analysis at different magnifications.

## Results

### Successful Targeting of the Gng5 Locus

**Figure 2:**
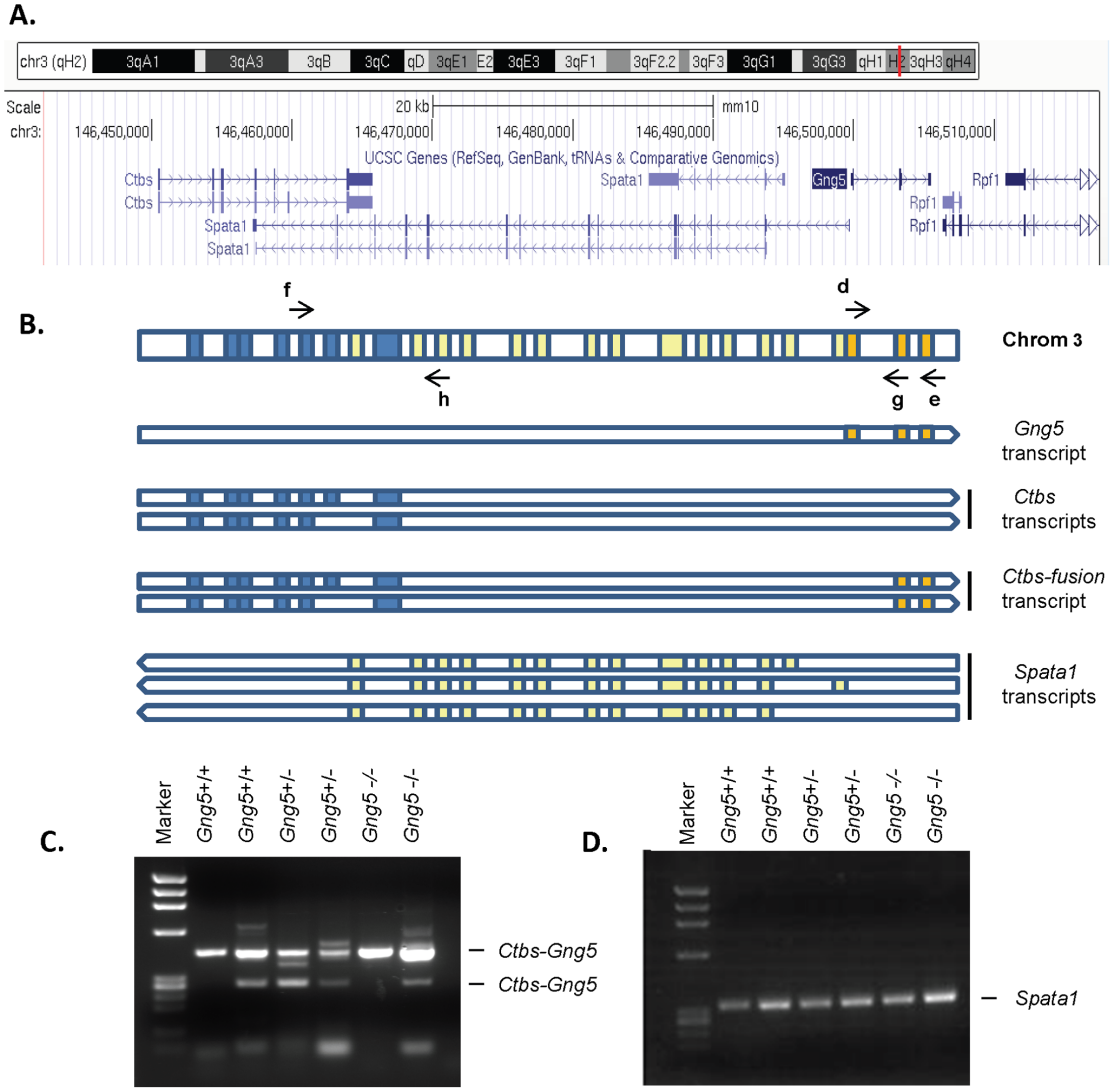
Successful targeting of the *Gng5* locus. **A.** Region of mouse chromosome 3∶146,110,000–147,170,000 containing the *Gng5* locus from the UCSC genome browser. **B.** The top bar of this schematic illustrates the arrangement of exons of *Gng5* (orange boxes), *Ctbs* (blue boxes), and *Spata1* (yellow boxes). The bars underneath illustrate the *Gng5* mRNA transcript, the two *Ctbs* splice variants, the two *Ctbs-Gng5* splice variants, and the three *Spata1* splice variants (not drawn to scale). Letters indicate RT-PCR primers which can be found in Table S1. **C.** RT-PCR of the *Ctbs-Gng5* fusion transcript (primers **f** and **g**) from the same embryos shown in Fig. 1E, confirming that expression of this fusion transcript is preserved in knockout embryos. Identification of the amplified products marked *Ctbs-Gng5* was confirmed by DNA sequence analysis (Figure S1). The first lane on both gels is a molecular weight marker, φX digested with HaeIII. **D.** RT-PCR of *Spata1* from the same embryos (primers **f** and **h**), demonstrating that expression of *Spata1* is preserved in knockout embryos.

Careful design of the targeting strategy was necessary to remove only exon 1 of *Gng5* and to minimize any impact on contiguous genes (Fig. 2A). The *Gng5* gene (orange blocks) lies within an intron of the chitobiase (*Ctbs*) gene (blue blocks) that produces two transcripts containing the last two exons of the *Gng5* locus (Fig. 2B) [37], [38]. Also, the *Gng5* gene resides in a head-head arrangement with the spermatogenesis-associated (*Spata1*) gene (yellow blocks) that generates multiple transcripts arising from different non-coding exons [39]. To confirm successful targeting of the *Gng5* locus, heterozygous mice were intercrossed to produce the three genotypes (*Gng5^+/+^, Gng5* ^+/−^, and *Gng5* ^−/−^ embryos). Using *Gng5* gene-specific primers (d,e; Fig. 2B), RT-PCR analysis confirmed the absence of *Gng5* transcript in knockout embryos (Fig. 1E). In contrast, all three genotypes showed similar expression of *Ctbs-Gng5* (Fig. 2C) and *Spata1* (Fig. 2D) transcripts, whose identities were confirmed by DNA sequence analysis (Fig. S1). Taken together, these results validate the targeting strategy by showing both loss of *Gng5* expression and preservation of expression from the two contiguous loci.

### Embryonic Phenotype

Heterozygous *Gng5* ^+/−^ intercrosses produced no homozygous *Gng5^−/−^* pups (Table 1). To determine when knockout embryos died, timed matings were set up and embryos collected at different gestational stages. Between embryonic days, e8.5 and e10.5, all three genotypes were present at the expected Mendelian frequency. However, all *Gng5^−/−^* embryos were severely compromised or dead by e10.5 (Table 1) and were readily identifiable by their morphologic defects that included abnormal headfolds, hypoplastic pharyngeal arches, and severe cardiac defects.

**Table 1:** Genotype distribution of embryos from *Gng5+/−* intercrosses.

Age		Number of pups or embryos observed (expected)			X^2^value	P value
	Total	+/+	+/−	−/−		
Postnatal Pups	89	32(22)	57(44)	0(22)	29.8	3.5×10^−7^
E8.5	44	7(11)	27(22)	10(11)	2.68	0.26
E9.5	48	18(12)	19(24)	11(12)	4.13	0.13
E 10.5	36	5(9)	21(18)	10(9)	2.39	0.30

**Figure 3:**
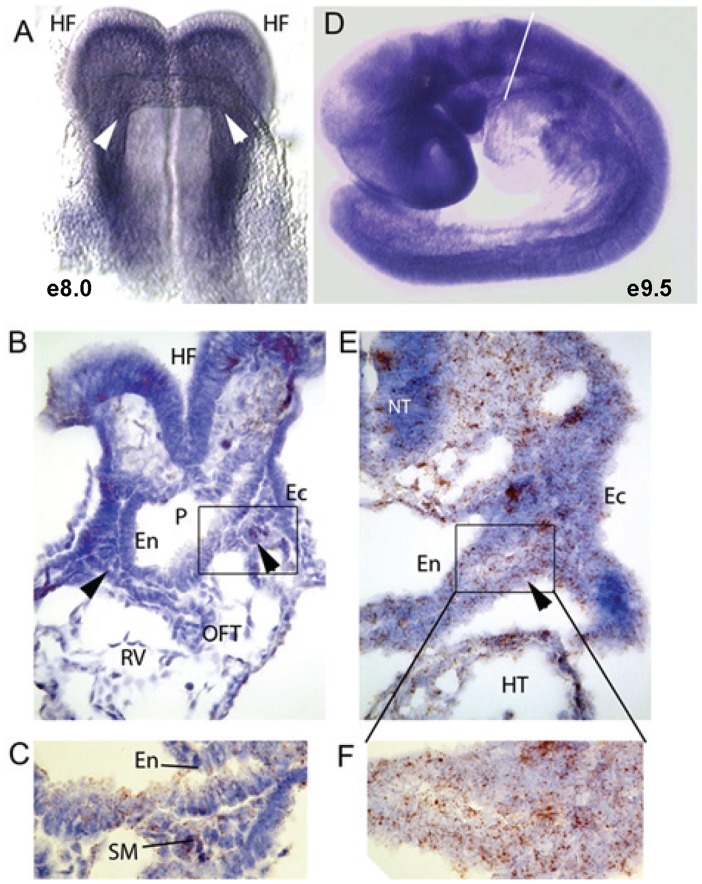
Developmental expression of *Gng5*. **A,** Ventral view of e8.0 mouse embryo after whole mount RNA *in situ* hybridization (blue signal) to detect *Gng5* mRNA. White arrowheads denote location of cardiac progenitors. HF, head fold. **B,** Section of e8.5 embryo through the region of the pharynx. Brown signal indicates *Gng5* transcripts in HF, endoderm (En), ectoderm (Ec) and cardiac progenitors (black arrowheads). There is little signal in the heart proper (RV, right ventricle; OFT, outflow tract). **C,** Magnification of the region boxed in B containing cardiac progenitors in splanchnic mesoderm (SM). **D,** Left lateral view of e9.5 mouse embryo after whole mount RNA *in situ* hybridization (blue signal) to detect *Gng5* mRNA. Widespread expression is present with less in the heart (white line denotes dorsal inflow region). **E,** Section of e9.5 embryo through the region of the pharynx. Brown signal indicates *Gng5*, endoderm (En), ectoderm (Ec) and cardiac progenitors (black arrowhead). There is also some signal in the heart (HT). **F,** Magnified view of pharyngeal and splanchnic mesoderm corresponding to boxed region in E.

To better understand the embryonic requirement for the G-protein γ_5_ subunit, we performed RNA *in situ* hybridization on whole or sectioned embryos (Fig. 3). In e8.0 whole embryos, *Gng5* transcript was broadly distributed in the anterior portion of the embryo (Fig. 3A). Of particular interest, the *Gng5* transcript was detected in cardiac precursors residing in the cardiac crescent (white arrowheads). Attesting to the specificity of the hybridization signal, no *Gng5* transcript was detectable in knockout embryos (Fig. S2). Next, we employed the RNAscope method to visualize the *Gng5* transcript in sectioned embryos. Compared to standard *in situ* RNA hybridization, this method is more sensitive and more quantitative since the amount of chromagen is directly correlated with the number of transcripts in each cell [40]. Sectioned embryos confirmed expression in cardiac precursors (Fig. 3B, black arrowheads), and also in the adjacent pharyngeal epithelia (boxed region magnified in Fig. 3C). In e9.5 whole embryos, *Gng5* transcript continued to be expressed throughout the embryo although levels in the heart proper were relatively lower than other regions (Fig. 3D). Sectioned embryos again confirmed *Gng5* expression in the cardiac precursors residing in the splanchnic mesoderm dorsal to the heart (Fig. 3E, black arrowhead and boxed region magnified in Fig. 3F). Thus, *Gng5* transcripts are found in regions relevant to mouse cardiogenesis.

### Defective Cardiogenesis

**Figure 4:**
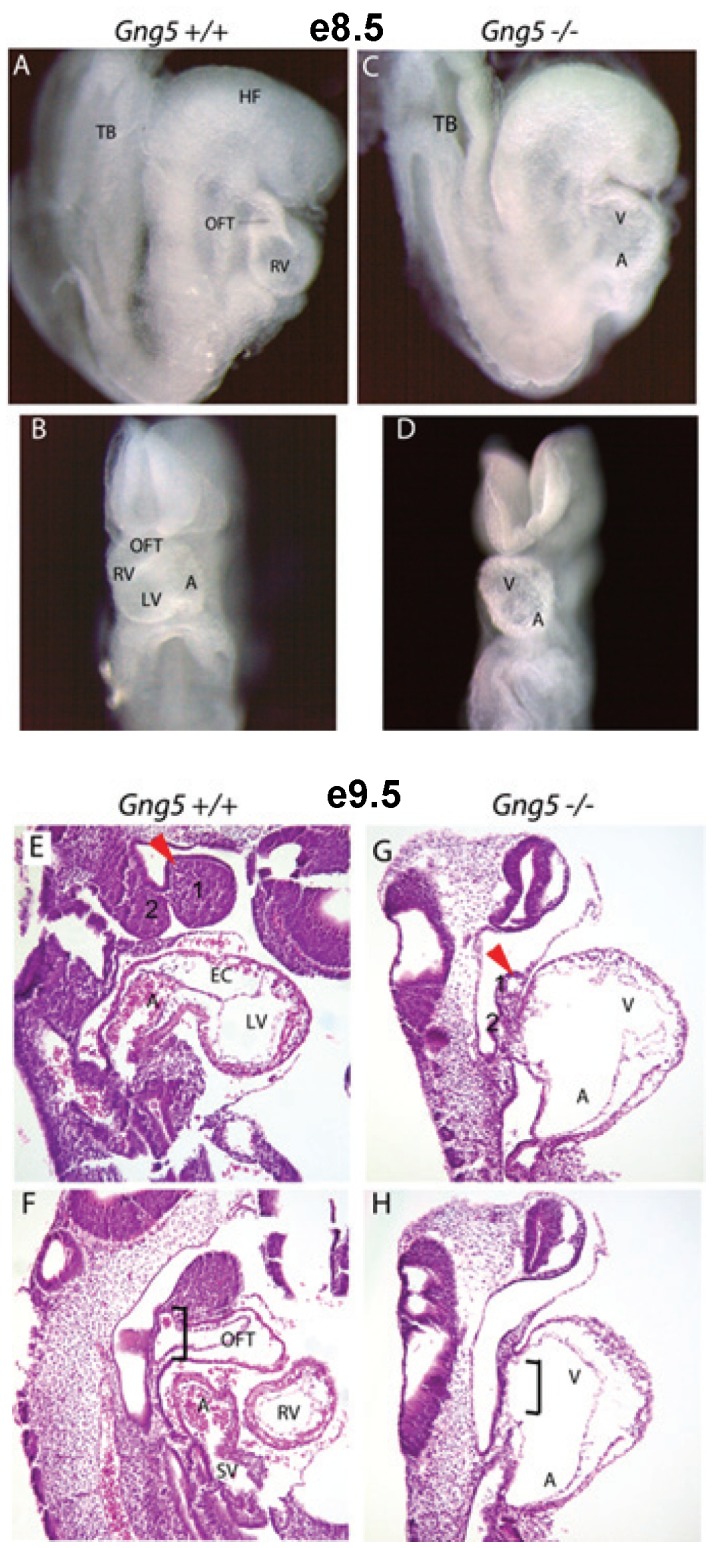
*Gng5^−/−^* mutants fail to form the cardiac outflow tract and right ventricle and have severely hypoplastic pharyngeal arches. **Panels A–D** images of e8.5 whole mount wild type and mutant embryos. By comparing the left lateral (A, C) and ventral (B, D) views of intact wild type and *Gng5^−/−^* embryos, respectively, the unlooped heart tube is clearly evident (B, D). **Panels E–H**, images of e9.5 wild type and mutant embryos. The left parasagittal section from a control embryo shows the inflow tract and left side of common atrium, endocardial cushion in the atrioventricular canal, and left ventricle (E). The red arrowheads mark the first pharyngeal arch, also labeled 1. The midline section shows the outflow tract connecting to aortic sac in the second pharyngeal arch (bracket), right ventricle, the right portion of the common atrium, and the sinus venosus in the control embryo (F). The left parasagittal section of a *Gng5^−/−^* mutant shows severely hypoplastic but vascularized first pharyngeal arch (red arrowhead), dilated heart tube with atrial chamber caudal to ventricle, narrow inflow, and paucity of cells in the pharyngeal mesoderm (G). The midline section shows the unlooped, dilated heart tube and no outflow tract; cardiac chamber opens directly into dilated aortic sac (bracket) in a *Gng5^−/−^* embryo (H). TB, tail bud; HF, head fold; OFT, outflow tract; RV, right ventricle; LV, left ventricle; A, atrium V, ventricle; EC, endocardial cushion; SV, sinus venosus.

At e8.5, morphologic analysis of *Gng5^−/−^* embryos revealed a specific set of anatomic defects, including severely hypoplastic pharyngeal arches and an unlooped cardiac tube (n = 25/25), which were never observed in their littermate controls (n = 17/17) (Fig. 4 A–D). Since an impaired cardiovascular system is the most common cause of death at this stage [41], we examined the overall structure of the heart and vasculature in more detail. At e9.5, histologic examination of control littermates revealed fully looped hearts characterized by four, primitive chambers along with distinct inflow and outflow tracts (n = 60/60) (Fig. 4 E,F). However, knockout littermates had unlooped hearts characterized by a primitive atrium caudal to a single ventricle with no demonstrable outflow tract (n = 16/16) (Fig. 4 G, H). These results demonstrate for the first time that *Gng5* disruption produces embryonic lethality reflecting an essential role for the G-protein γ_5_ subtype in right ventricle (RV) and outflow tract (OFT) formation.

**Figure 5:**
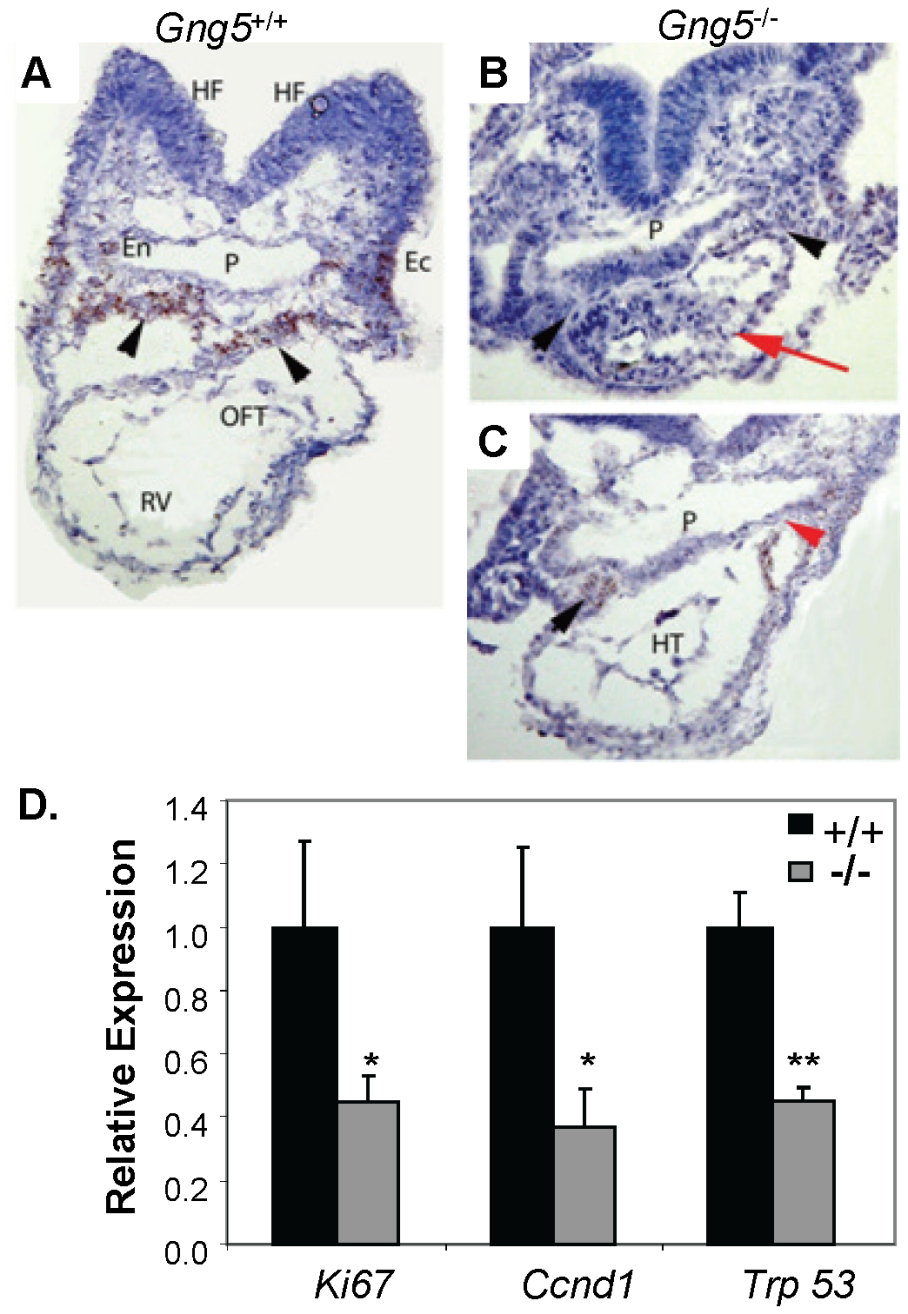
*Gng5^−/−^* mutants show loss of second heart field and reduced expression of proliferative markers. **Panels A–C**, control and mutant embryos stained for *Fgf8* expression in the second heart field. As shown by *in situ* hybridization for *Fgf8* transcripts, the e8.5 control embryo (A) displays numerous pharyngeal mesodermal cells exhibiting *Fgf8* expression (brown staining; black arrowheads). These cells represent progenitors of the right ventricle (RV) and outflow tract (OFT) that reside dorsal to the heart. *Fgf8* expression is also detected in the lateral pharyngeal endoderm (En) and pharyngeal ectoderm (Ec). In stark contrast, The *Gng5^−/−^* mutants (B,C) have fewer pharyngeal mesoderm cells and reduced *Fgf8* expression in this region. The red arrow (B) indicates the most rostral portion of heart tube and the absence of OFT; the black arrowhead denotes absent *Fgf8* expression in the thin layer of pharyngeal mesoderm dorsal to the heart. In a more caudal section (C), the heart tube (HT) is visible and faint *Fgf8* expression is detected in the mesoderm adjacent to the heart tube (black arrowhead) and in the most proximal portion of the left side of the heart tube and adjacent mesoderm (red arrowhead). HF, head fold, P, pharynx; En, endoderm; Ec, ectoderm. **Panel D**, relative expression of proliferative markers in pharyngeal region of e9.0 control and *Gng5* ^−/−^ mutant embryos. As shown by qPCR analysis, *Gng5^−/−^* mutant embryos display reduced expression of three proliferative markers in the second heart field (* *p*<0.002; ** *p*<0.0001 by Student’s *t*-test), using elongation factor 1 (*Eef1a1)* as the housekeeping gene. All primer sequences can be found in Table S1.

To probe the basis for this phenotype, we assessed the integrity of the second heart field normally giving rise to RV and OFT formation [42]. Integral to this process, fibroblast growth factor signaling (*ie*, Fgf8/Fgf10) drives continued proliferation of cardiac precursor cells within the pharyngeal mesoderm that are required for RV and OFT formation [36], [43]–[47]. Using the RNAscope procedure [40]. we compared *Fgf8* expression within the pharyngeal mesoderm of control and knockout embryos sectioned transversely (Fig. 5A–C). At e8.5, the control embryo exhibited intense *Fgf8* expression in the numerous cardiac precursor cells within the pharyngeal mesoderm (Fig. 5A, brown staining, black arrowheads), as well as other cells within the pharyngeal endoderm and ectoderm. In contrast, *Gng5^−/−^* littermates showed only faint *Fgf8* expression reflecting both fewer numbers of cardiac precursor cells and markedly less chromagen present in the remaining cells (Fig. 5B–C, brown staining, black arrowheads). In the top panel of the mutant (Fig. 5B), the red arrowhead labels the rostral portion of the heart tube and the absence of OFT. In the bottom panel (Fig. 5C), the red arrowhead labels more caudal mesoderm adjacent to the left side of the heart tube (Fig. 5C). Taken together, these data demonstrate that loss of the G-γ_5_ subtype is associated with a defect in the second heart field, which is consistent with both the nature and severity of the cardiac defects observed in *Gng5^−/−^* embryos.

**Figure 6:**
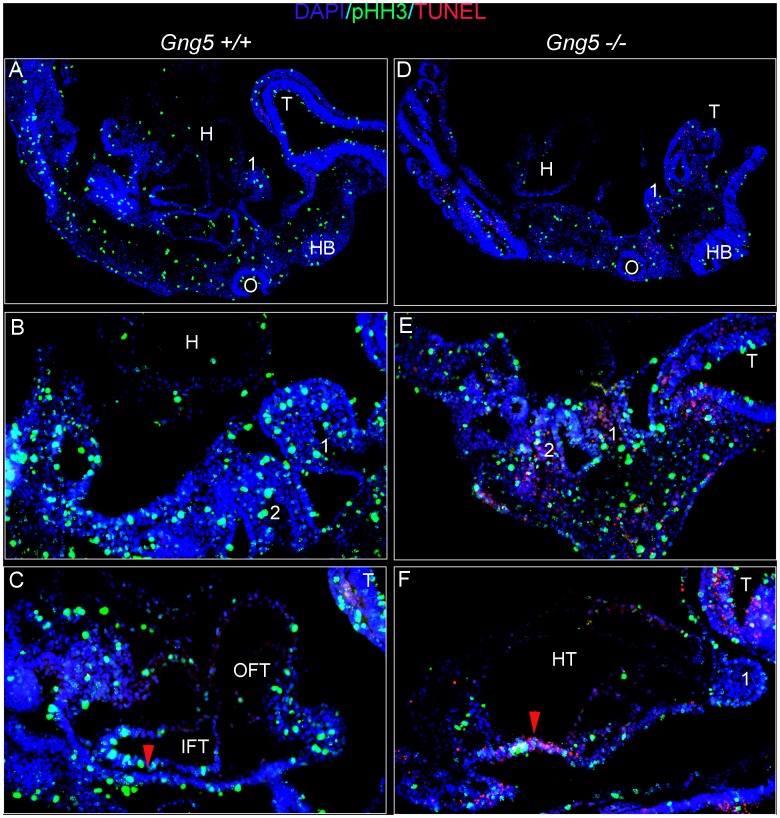
*Gng5^−/−^* mutants have decreased cell proliferation and survival. Sagittal cryosections of e9.5 control and mutant embryos after immunohistochemical staining for DNA (DAPI, blue), cells in mitosis (anti-pHH3, green), and cells undergoing apoptosis (TUNEL, red). In all panels, rostral is at right, ventral at top. **Panels A, D** represent 10X views of control (A) and mutant (D) embryos, while **Panels B,C,E,F** show 20x views of control (B,C) and mutant (E,F) sections. The red arrowheads in C and F denote second heart field pharyngeal mesoderm and adjacent endoderm. The pharyngeal arches are numbered. H, heart; T, telencephalon; HB, hindbrain; O, otocyst; OFT, outflow tract; IFT; inflow tract; HT, heart tube (mutant only).

To characterize the molecular events underlying this defect, we examined gene expression in pharyngeal tissues encompassing the second heart field that had been microdissected from control and mutant embryos (Fig. 5D). Since sustained proliferation of cardiac precursor cells within this region is critical for RV and OFT formation [42], we first assessed cell proliferation by determining the relative levels of several proliferative markers [48], [49] in pharyngeal tissues from control and mutant embryos. Notably, all three proliferative markers were significantly reduced in mutant pharyngeal tissues (Fig. 5D). Next, we assayed cell proliferation and apoptosis by immunohistochemical staining of control and mutant embryos (Fig. 6). Cryosections were stained for DNA (DAPI, blue), cells in mitosis (anti-PHH3, green), and cells undergoing apoptosis (TUNEL, red) [36], [45]. This analysis revealed globally decreased proliferation in *Gng5^−/−^* embryos and multiple regions of abnormal apoptosis. Although no apoptosis was detected in the heart tubes of the mutants, nearly 10% of cardiomyocytes in control heart showed anti-pHH3 staining, whereas only occasional proliferating cells were present in the hearts of mutants. Even more striking, the second heart field mesoderm lying dorsal to the heart exhibited both decreased cell proliferation and increased cell death in the mutants (Fig. 6C versus D). Taken together, these results identify a novel role for the G-γ_5_ subtype in regulating the expansion and/or survival of cardiac precursor cells. Consistent with previous reports showing enriched *Gng5* expression in neural stem cells [32], [33], our data also support a similar role in other cell populations, including neural progenitor cells.

## Discussion

Homozygous disruption of the *Gng5* gene encoding the G-γ_5_ subtype produces a complex phenotype characterized by severe defects in the head, heart, and other developing structures (Fig. 4). In this paper, we focus on its requirement in cardiac development since the impaired cardiac performance limits our ability to examine any direct role in brain development. Notably, we found that all mutant embryos have severe cardiac defects that are incompatible with survival (Table 1). The observation that e8.5 mutant embryos have an overtly normal linear heart tube suggests normal specification and deployment of precursor cells from the first heart field [50], although further analyses of this structure will be needed to rule out any molecular or functional changes. In contrast, both looping of the heart tube and formation of the RV and OFT are notably absent in e9.5 knockout embryos. Our subsequent studies confirmed loss of G-βγ_5_ signaling disrupts the expansion and/or survival of cardiac precursor cells that contribute to chamber specification and OFT formation [42].

### Developmental Function

How G-γ_5_ influences the number of cardiac precursor cells is not entirely clear. Multiple signaling cascades ensure the proper balance between cellular proliferation, differentiation, and survival [42], [46]. Of these, Fgf8 is one of the most important factors involved in this process [46]. Therefore, the finding that G*ng5^−/−^* mutants have fewer *Fgf8* expressing precursor cells in the pharynx and diminished *Fgf8* expression in the few remaining cells (Fig. 5) offers a causal basis for this phenotype. Cross talk between G-protein and growth factor signaling cascades could provide a means of amplifying intracellular second messengers required for proliferation and/or survival of cells [11], [12]. By analogy to vascular endothelial growth factor [51], the mechanism could involve G-protein mediated induction of *Fgf8* expression or transactivation of Fgf receptor signaling. Alternatively, intersection between G-protein and extracellular matrix receptor mechanisms could offer a means of transducing mechanical signals responsible for coordinating cell adhesion and proliferation [9], [10]. The mechanism could involve an interaction between the G-γ_5_ subunit and integrin receptor since both localize to focal adhesion complexes [34] and both show similar loss-of-function phenotypes [52]. Accordingly, we hypothesize that G-βγ_5_ signaling may represent a point of convergence for G-protein-coupled, growth factor, and extracellular matrix receptors that control expansion or survival of cardiac precursor cells.

### Irreplaceable Role for G-γ_5_ in Cardiac Development

**Figure 7:**
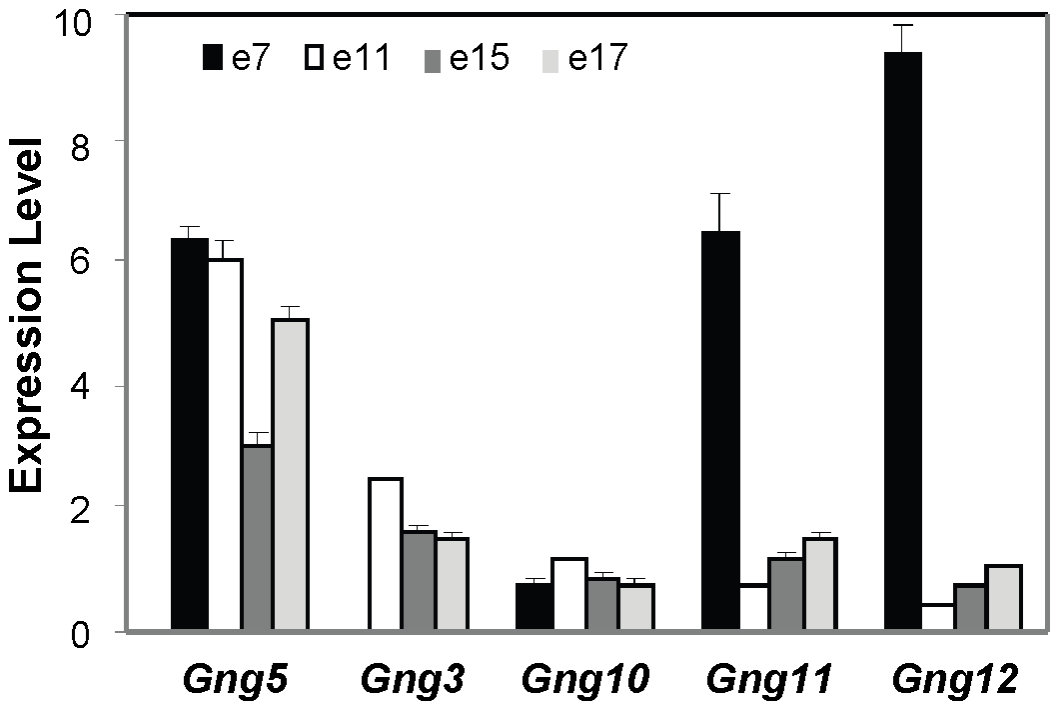
Non-redundant function of *Gng5* gene during development. As shown by qPCR analysis on a normalized mouse cDNA panel containing different gestational stages (Mouse MTC Panel1, Clontech), multiple *Gng* family members are expressed at developmental stages relevant to neural and cardiac development. All primer sequences can be found in Table S1.

The complete penetrance of the *Gng5^−/−^* phenotype demonstrates the cardiogenic function of the G-γ_5_ subtype cannot be replaced by other members of the G-γ subunit family. At this stage, we can only speculate as to the basis for its unique requirement. One possibility is that the G-γ_5_ subtype is exclusively expressed in early embryogenesis. However, arguing against this possibility, the *Gng5, Gng11*, and *Gng12* transcripts are all found to be abundantly expressed in the period (Fig. 7) immediately preceding the appearance of cardiac defects in *Gng5^−/−^* embryos (Fig. 4). Another possibility is that these three G-γ members are sequestered between different cell lineages or subcellular compartments. Providing some support for this possibility, the G-γ_5_ protein is enriched in focal adhesions that represent subcellular sites responsible for coordinating growth and adhesion [34]. Although the basis for its unique localization is not known, variable post-translational processing of the G-γ_5_ protein could offer a potential mechanism [53], [54]. In contrast to other family members, the majority of the G-γ_5_ protein retains the carboxy-terminal, CAAX motif. Since this motif represents a potential PDZ binding protein site [55], directed protein-protein interactions could account for targeting of a specific G-αβγ_5_ heterotrimer to focal adhesions, and hence, its unique requirement in cardiac development.

At this stage, the identity of the G-αβγ_5_ heterotrimer that functions in the context of cardiac progenitor cells is not known. None of the sixteen *Gna* subunit genes that have ablated in mice [17] recapitulate the cardiac defects seen upon disruption of the G-γ_5_ subtype. This suggests that the G-βγ_5_ dimer performs a separate role above and beyond that of its G-α partner in this process. Providing additional support for this possibility, genetic ablation of the *Drosophila* ancestral G-γ subunit also blocks heart development [56], [57]. Likewise, neither the *Gnb1 or Gnb5* genes that have targeted in mice [22], [23] phenocopies the cardiac defects observed upon loss of the G-γ_5_ subtype. This implies that one of the three remaining G-β_2_, β_3_, or β_4_ subtypes partners with the G-γ_5_ protein, or that the closely related G-β subtypes can substitute for one and another in this particular context.

### Clinical Relevance

Congenital heart defects, including OFT malformations, occur in >1% of live births [58], [59]. Thus, the identification of G-βγ_5_ signaling as a major player in this process is translationally significant since successful intervention will only come from a better understanding of the signaling cascades driving OFT formation. This finding could also be clinically relevant since statins block the function of the G-γ subtypes [53], [56] and inadvertent use of statins during pregnancy is reportedly associated with increased incidence of infants with head and heart defects [60]. In fact, statins produce cardiac defects that are phenocopied by genetic ablation of the ancesteral *gng* gene in flies [56], [57]. Assuming the *Gng5* gene performs a similar role in mammals, our results could be important in guiding the use of statins that are increasingly being administered to women of child-bearing age [61].

## Supporting Information

Figure S1 **Preservation of the** ***Ctbs*** **locus.** As confirmed by DNA sequence analysis of amplified PCR product, *Ctbs-Gng5* transcripts are expressed in e9.5 knockout embryos even though *Gng5* transcripts are lost (Fig. 1E).
(TIF)

Figure S2 **Validation of** ***in situ*** **RNA hybridization procedure.** *Gng5* transcripts are widely expressed in anterior portion of e8.5 wild type embryo (left panel). Attesting to the specificity of signal, no staining is observed in stage-matched, knockout embryo (right panel).
(TIF)

Table S1 **List of PCR primers.** Gene-specific expression was detected using indicated primer pairs.
(TIFF)
