# Supplementary figures and images for: Disruption of G-Protein γ5 Subtype Causes Embryonic Lethality in Mice
Source: PLoS One. 2014 Mar 5;9(3):e90970. doi: 10.1371/journal.pone.0090970 (PMC3944967; doi:10.1371/journal.pone.0090970)

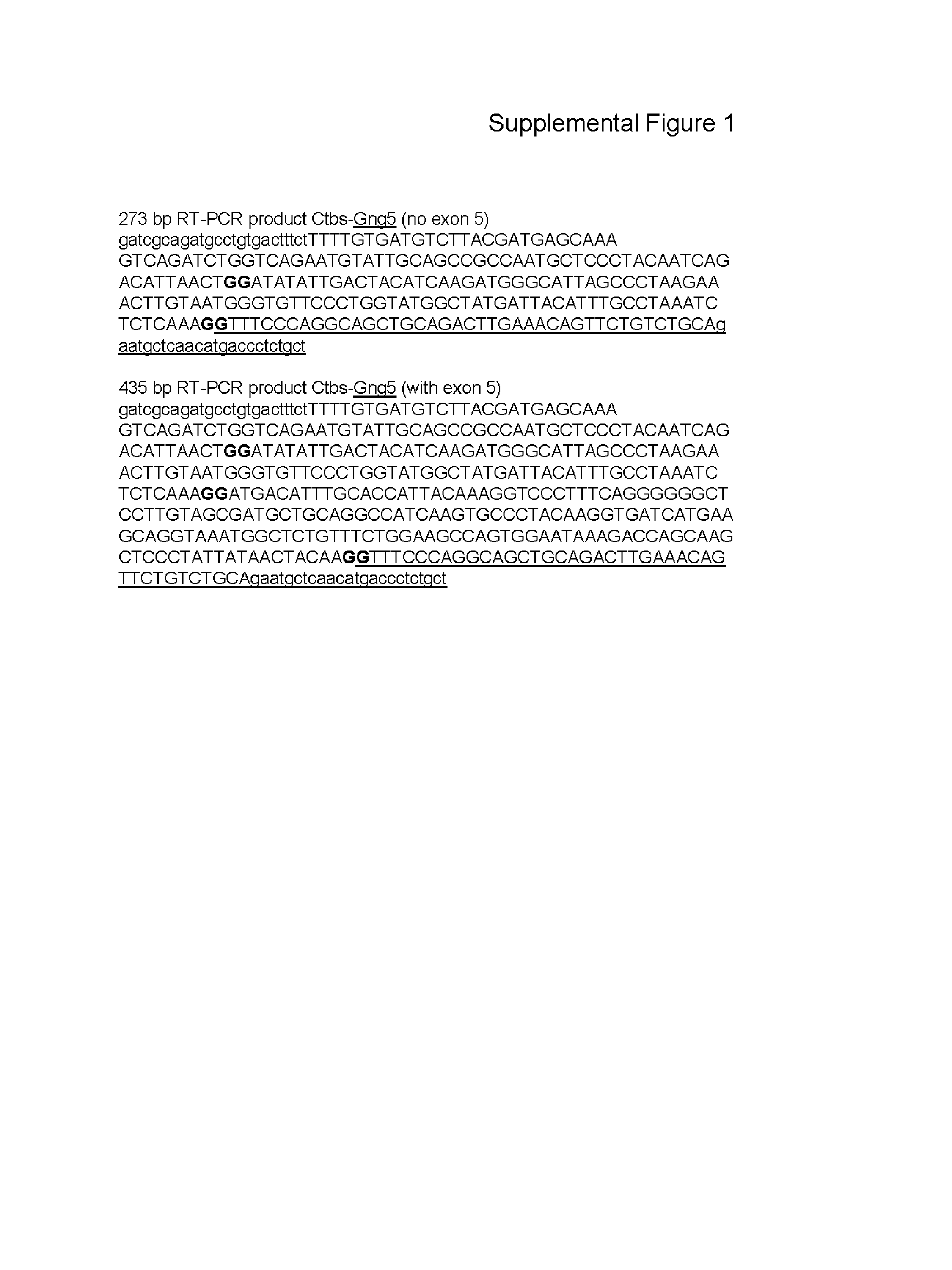

Supplement: Figure S1 — Preservation of the Ctbs locus. As confirmed by DNA sequence analysis of amplified PCR product, Ctbs-Gng5 transcripts are expressed in e9.5 knockout embryos even though Gng5 transcripts are lost (Fig. 1E). (TIF) [file pone.0090970.s001.tif]

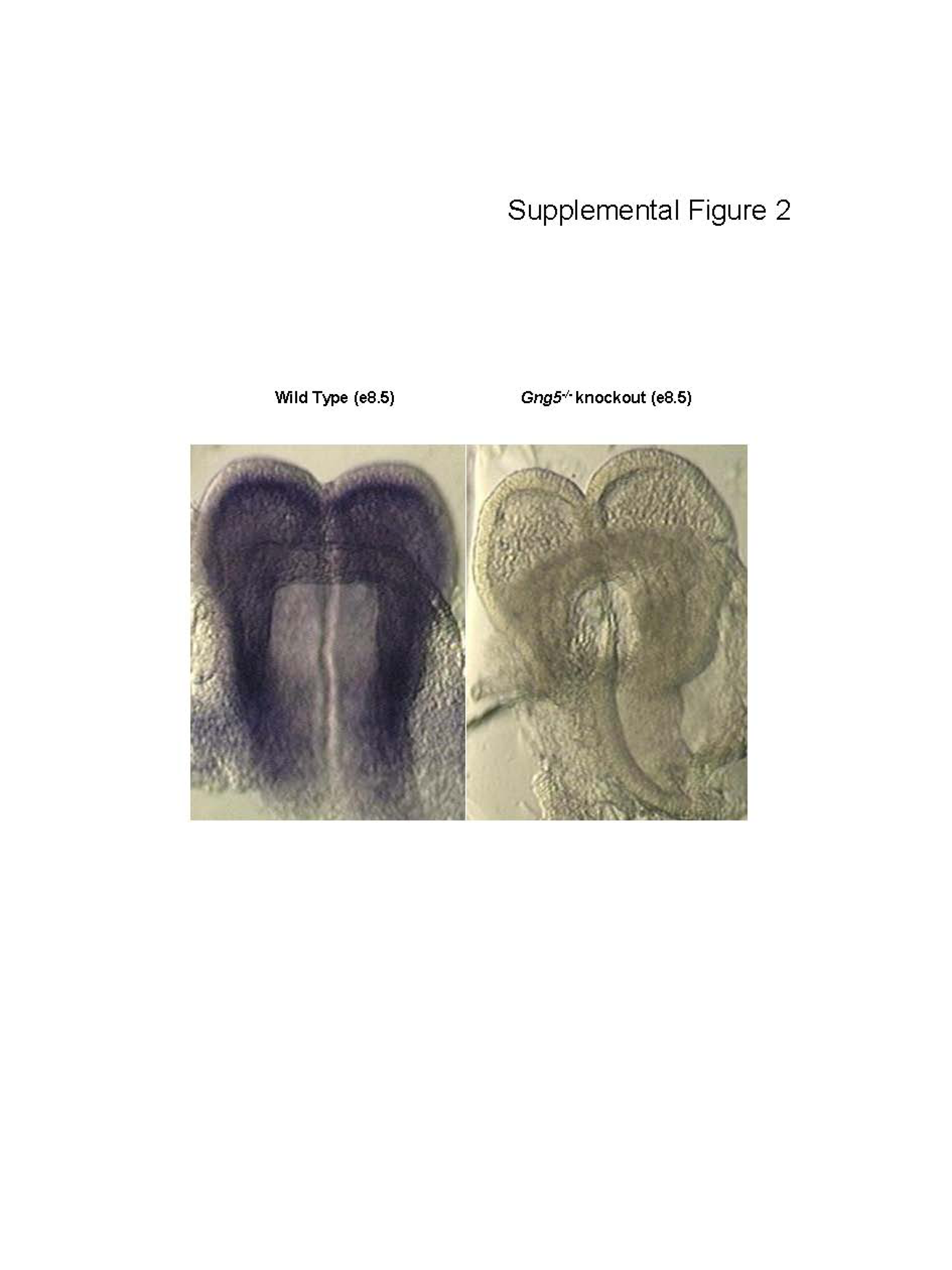

Supplement: Figure S2 — Validation of in situ RNA hybridization procedure. Gng5 transcripts are widely expressed in anterior portion of e8.5 wild type embryo (left panel). Attesting to the specificity of signal, no staining is observed in stage-matched, knockout embryo (right panel). (TIF) [file pone.0090970.s002.tif]
